# Thyroid Hormone Nuclear Receptor TRα1 and Canonical WNT Pathway Cross-Regulation in Normal Intestine and Cancer

**DOI:** 10.3389/fendo.2021.725708

**Published:** 2021-12-10

**Authors:** Maria Sirakov, Leo Claret, Michelina Plateroti

**Affiliations:** ^1^ Department of Biology and Evolution of Marine Organisms, Stazione Zoologica Anton Dohrn, Naples, Italy; ^2^ Université de Strasbourg, Inserm, Interface de Recherche fondamentale et Appliquée en Cancérologie (IRFAC)/Unité Mixte de Recherche (UMR)-S1113, Fédération de Médecine Translationnelle de Strasbourg (FMTS), Strasbourg, France

**Keywords:** thyroid hormones, thyroid hormone receptors, intestinal epithelium, intestinal carcinogenesis, canonical WNT pathway

## Abstract

A pivotal role of thyroid hormones and their nuclear receptors in intestinal development and homeostasis have been described, whereas their involvement in intestinal carcinogenesis is still controversial. In this perspective article we briefly summarize the recent advances in this field and present new data regarding their functional interaction with one of the most important signaling pathway, such as WNT, regulating intestinal development and carcinogenesis. These complex interactions unveil new concepts and will surely be of importance for translational research.

## Introduction

The role of the Thyroid Hormones (THs) in intestinal development have been established since the beginning of the 20^th^ century based on the observations on amphibian metamorphosis. Indeed, during this postnatal developmental process the gastrointestinal tract is dramatically remodeled, with a first phase of apoptosis followed by a strong increase of cell proliferation correlating with an increase of circulating THs (1).

## The Intestinal Epithelium

The intestinal epithelium is a tissue which combines the absorptive properties of a single layered/high-surface epithelium with the protective advantages of a constantly renewing barrier. It comprises six different mature cell types, which are separated into absorptive (enterocytes and M cells) and secretory (goblet, enteroendocrine, tuft and paneth cells) lineages. The different cell types allow functions such as the nutrient uptake, metabolic control and immune regulation. Thus, the intestinal epithelium is a multifunctional dynamic tissue highly dependent on continuous supply of all cell types in appropriate ratios (2). The epithelial lineages are derived from Intestinal Stem Cells (ISCs) which are located at or near the base of the intestinal crypts (3). The crypts are invagination of the intestinal wall, where the ISCs are well protected from the hazards of the digestive process occurring in the lumen. Within the crypts, self-renewing ISCs give rise to progenitor cells that rapidly proliferate and commit into epithelial lineages (2, 3). These cells are then pushed out of the crypts, they differentiate while migrating and die by apoptosis at the apex of the villi. This system efficiently secludes the ISCs and exposes to the hazards of the digestive tract only postmitotic cells that are programmed to die (2, 3). Paneth cells represent the exception to this rule, since they migrate downwards to the base of the crypts where they reside with a lifespan of approximately 30 days (2).

Homeodomain transcription factors (such as *Cdx* genes) and several pathways (including WNT, Hedgehog, Notch, BMP) play crucial roles in diverse developmental and homeostatic processes (2, 4). While the molecular basis of their action is well understood, the complex cross-regulation that occurs between them and/or with the environment (*i.e.* fibroblasts, myofibroblasts, immune cells, microbiota) still need to be better defined.

## From Homeostasis to Cancer: The Canonical WNT Pathway in the Intestine

Among the signalling pathways mentioned in the previous paragraph, the WNT pathway is highly conserved and fundamental for intestinal development, cell proliferation and differentiation, ISCs maintenance. No other pathway plays such an important role on the self-renewal/proliferative capability and cell fate of ISCs (2, 5). Indeed, the *Tcf7l2*-knockout mice (the *Tcf7l2* gene encodes for TCF4, the transcriptional activator of the canonical WNT signalling) show a strong impairment in epithelial renewal and the ISC compartment is entirely absent (6). Other two papers reported that blocking WNT pathway by the targeted overexpression of the WNT inhibitor Dickkopf-1 in the intestinal epithelium, induces *in vivo* the complete loss of the proliferative compartment (7, 8). On the other side, van de Wetering and colleagues concluded that the β-catenin/Tcf4 transcriptional complex constitutes the master switch that controls proliferation versus differentiation in healthy and malignant intestinal epithelial cells (9). In the context of cancer, the WNT signalling pathway is well known for its role as a key driver of intestinal tumorigenesis. In fact, among the most frequently mutated genes, the very early event in cell transformation depends on the uncontrolled activation of the WNT/β-catenin pathway. This is driven very frequently by loss-of-function (LOF) mutations of the *APC/Apc* gene, whose product in a wild type (WT) context, blocks the functionality of the β-catenin and thus the activity of the pathway (10, 11). Other genes participating in this pathway frequently mutated in cancer include *AXIN2/Axin2* (LOF) as well as *CTNNB1/Ctnnb1* gain-of-function (GOF), the last encoding for the β-catenin protein. Specifically, *CTNNB1*/*Ctnnb1* mutations are responsible for a stabilized oncogenic form of the protein (10, 11). It is important to underline that several signalling pathways can synergize with and induce WNT pathway activation to accelerate the early steps of tumorigenesis. Indeed, the Notch (12), Shh (13), Yap/Taz (14) are examples of signalling pathways which trigger APC-dependent tumorigenesis. Finally, we have demonstrated that THs *via* their Nuclear Receptor α1 (namely Thyroid hormone Receptor α1, TRα1) can be included in this list (15, 16).

## TRs in the Intestinal Development and Homeostasis

The action of THs in the nucleus depends on the ability of TRs to bind the hormone T3, which is then considered as the active hormone and the cellular effector of THs. TRs activate or repress the transcription of target genes by binding to specific DNA sequences called thyroid hormone response elements (TREs) (17). During the amphibian metamorphosis different TRs are distinctly involved. Indeed, the Thyroid hormone Receptor α (TRα) is expressed at low level in the tadpole pre-metamorphic intestine whereas the Thyroid hormone Receptor β (TRβ) is strongly increased after the surge in the TH level and they both play a fundamental role during the gut remodeling process (18). Moving to mammals, the generation of specific murine models helped to elucidate the function of THs in multiple organs and tissues including the intestinal epithelium (19). In mammals, the postnatal maturation consists of an increase in mucosal growth coupled to a burst in cell proliferation (19). Interestingly, THs level increases significantly in rodents at the weaning period (20), when the intestine undergoes this structural and functional reshaping. This increase has been also correlated with the mucosal growth and the onset of adult-type digestive enzymes expression in the enterocytes (21). Detailed analyses in murine models helped to define the different specific actions of TRα1 in intestinal epithelium progenitor/stem cell physiology. First, their proliferation strongly correlates with T3 levels and TRα1 expression (21, 22). Second, the activity of the ISCs at homeostasis and the regenerative properties of the epithelium after γ-ray irradiation are strongly affected by the lack of TRα1 expression (23, 24). Third, the targeted overexpression of TRα1 in the intestinal epithelium (*vil*-TRα1 mice) results in crypt hyperplasia and enhanced proliferation up to adenoma development (15).

## THs-TRs and Colorectal Cancers: Still More Work Needed

Epidemiologic studies attempted to define a correlation between altered THs status in patients and cancer development (25). Generally speaking, there is still a lack of consensus since both a stimulating/blocking action of THs or mutated TRs have been described in several tumor types (25–28). Accordingly, hypothyroidism is a beneficial factor for ocular melanoma and mammary cancer (29) whereas it represents an aggravating risk in the case of hepatocarcinomas (26). Nevertheless, an increased risk for colon, lung, prostate, and breast cancer with increased THs has been demonstrated, even suggesting a TH-dose effect on cancer occurrence (30). The ambiguity from epidemiological studies on THs and colorectal cancer (CRC) may arise from important missing information on: (*i*) mutational status of oncogenes (*APC*, *KRAS*, *TP53*) when hypo- or hyper-thyroidism appears, (*ii*) combination of mutations and/or expression of key genes and/or activation or repression of signalling pathways in different cell types influencing the biology of the cancer cells, (*iii*) local availability of the hormone (31) which is not directly correlated with the circulating levels of the THs but it is dependent on the levels of the TH-activating/inactivating enzymes and transporters (32). Indeed, the deiodinases selenoenzymes such as Dio2 (which converts T4 into T3), Dio3 (which converts T4 and T3 into revT3 and T2) appear important actors of THs activity (32). A complex interplay between Dio2 (*i.e.*, high T3), Dio3 (*i.e.* low T3) and Shh (33) or WNT (34) has been described in skin tumors or in colon cancer, respectively. In this last case, Dio3 is upregulated by WNT signal resulting in a positive effect on cell proliferation (34) and, contrary to our observations in animals (15, 16, 24), a report also indicates a negative effect of T3 on cancer stem cells maintenance *in vitro* (35). Concerning the TRs, it has been shown that their mutation or aberrant expression is associated with gastrointestinal tumors (25). In particular, TRβ gene is frequently methylated and its expression strongly decreased in colon cancer (36) whereas it is still unclear whether in this same context *TRα* gene expression is altered.

More work is clearly needed. In particular, the studies reported above do not take into account the complexity and the heterogeneity of the tumors. Hypo- or hyper-thyroidism *per se* are probably not directly involved in inducing mutations, but stimulate or inhibit cellular processes that can facilitate tumor development in the presence of a favorable genetic background.

## TRα1/WNT Crosstalk in the Physio-Pathology of the Intestine

TRα1 controls the proliferation of the mouse intestinal epithelium precursor cells by modulating, directly or indirectly, the expression of genes involved in cell cycle control, some of them being related to the WNT pathway (20, 37). For instance, TRα1 is a direct transcriptional regulator of the *Ctnnb1* gene. The increased expression of β-catenin, in turn, activates its targets such as *cyclins D1* and *D2* as well as *c-Myc* which can be considered TRα1 indirect targets (20). Another TRα1 direct target in the intestinal epithelium is the secreted frizzled-related protein (*Sfrp2*), which is another component of the WNT pathway (38). Interestingly, through its regulation by TRα1, we showed that sFRP2 stabilizes β-catenin in intestinal progenitors *in vivo* and in primary cells (39). These genes have been identified through a transcriptional analysis performed in cells isolated from the intestinal crypts of WT, TRα^0/0^ and TRβ^-/-^ mice (37). Moreover, the constitutive TRα1 overexpression in the intestinal epithelium (*vil*-TRα1 mice) not only confirmed their regulation but showed an increased cell proliferation and adenoma development even though it was not able *per se* to induce tumorigenesis (15). Interestingly, the TRα1 overexpression enhanced the intestinal tumorigenic process in the Apc^+/1638N^ (40) animals (*vil*-TRα1/Apc^+/1638N^ mice, hereafter designated as *vil*-TRα1/Apc) (15) while TRα-KO in the same Apc-mutated background retarded tumor development (16). One of the most interesting molecular features of the *vil*-TRα1/Apc mice is the increased activity of the WNT pathway compared with that of the Apc-simple mutants, which is likely responsible of the earlier onset of tumor development in the *vil-*TRα1/Apc mice (15). Importantly, looking for the mechanisms involved, we characterized the molecular signature of TRα1-expressing murine tumors that includes Wnt and Notch pathways as well as stem cell markers (16). We also demonstrated in cohorts of CRC patients a significant up-regulation of TRα1 and a positive highly significant correlation between TRα1 expression and WNT pathway activity, therefore validating the relevance of the fundamental observations in clinics (16). Finally, the modulation of TRα1 expression in human colon adenocarcinoma cell lines, directly correlated with proliferation, migration and WNT pathway activity (41).

**Figure 1:**
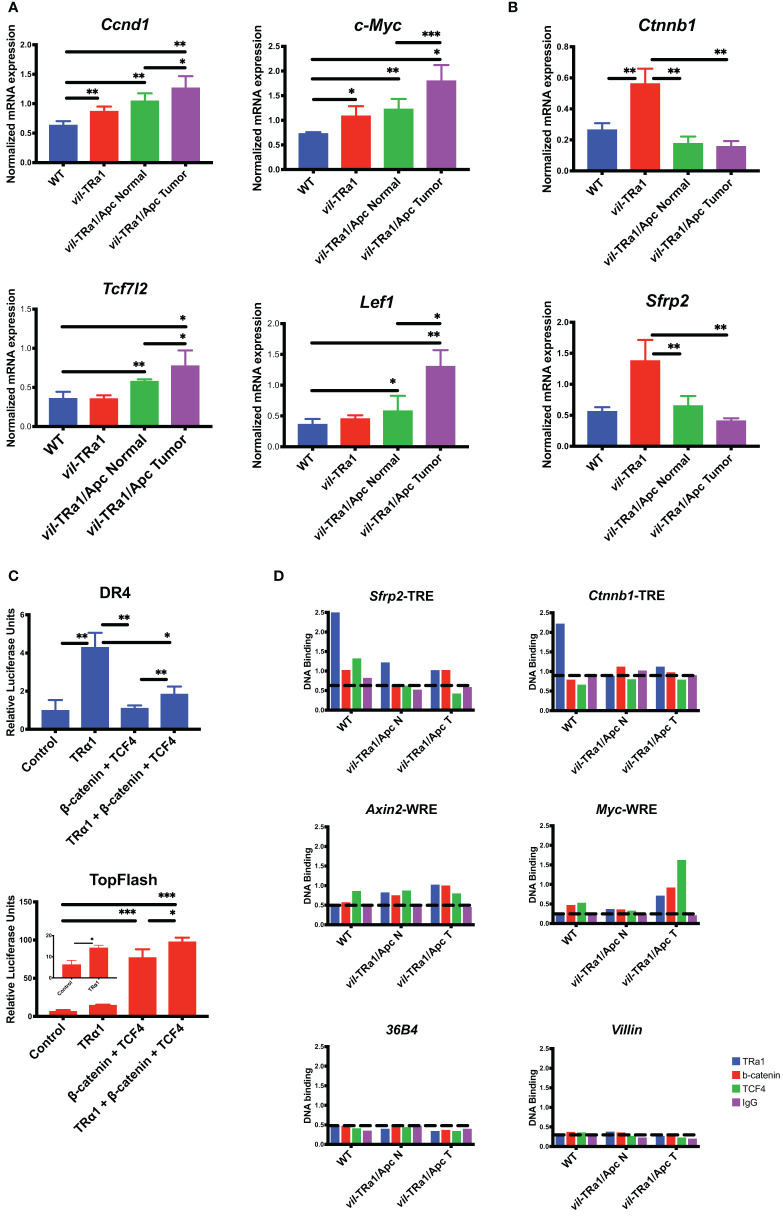
Functional interaction between TRα1 and β-catenin/Tcf4 complex. **(A, B)** Gene expression analysis was performed in the intestine or tumors from 1-month (young) or 6-month-old mice of the indicated genotype. Specifically, *Ccnd1* and *c-Myc* Wnt targets and, *Tcf7l2* and *Lef1* Wnt transcriptional effectors **(A)** or the TRα1 direct targets *Ctnnb1* and *Sfrp2* **(B)** were analyzed. Values represent fold change ± sd, after normalization with *Ppib*. *P < 0.05, **P < 0.01, ***P < 0.001 compared to indicated conditions, by unpaired two-tailed Student’s t-test (n=4). **(C)** A synthetic DR4-driving luciferase reporter (TRα1 response) or the TopFlash luciferase reporter (Wnt response) were transfected into Caco2 cells maintained in culture medium containing physiological concentrations of T3, together with TRα1, Tcf4 or β-catenin expression vectors in different combinations, as indicated. Histograms represent mean ± sd. *P < 0.05, **P < 0.01, ***P < 0.001, compared to indicated conditions, by unpaired two-tailed Student’s t-test (n=9). **(D)** ChIP analysis was performed on chromatin isolated from the intestine of WT or *vil*-TRα1/Apc mice normal intestine (N) or tumors (T). DNA/protein complexes were precipitated with anti-TRα1, anti-β-catenin, anti-Tcf4 antibodies or rabbit IgG (negative control). qPCR was performed on purified DNA from each condition by using specific primers covering the TRE of *Sfrp2* and *Ctnnb1*, the WRE of *Axin2* and c-*Myc* or the promoters of *Villin* and *36B4* as indicated; the *Ppia* gene was used as internal control. Histograms represent the specific-DNA enrichment in each sample precipitated with the indicated antibody. The horizontal black dotted bars in each panel delineates the threshold of binding specificity determined by the IgG non-specific binding.

In order to further investigate and shed more light in this cross-talk we performed cellular and molecular analyses in animals as well as in cells (methodological details in **Supplementary Materials and Methods**). As shown in **Figure 1**, the mRNA expression of WNT targets such as *Ccnd1* and *c-Myc* (**Figure 1A**) is up-regulated in *vil*-TRα1 intestines compared to the WT and their level is similar to the normal part of the *vil*-TRα1/Apc double mutant mice. However, in the lesions of these animals there is a further up-regulation, probably due to the WNT pathway hyperactivation in the context of the synergistic TRα1 overexpression and Apc mutation (15, 16). The mRNAs encoding for the WNT transcriptional effectors *Tcf7l2* and *Lef1* present an expression pattern similar to those of their molecular targets (**Figure 1A**). The expression of TRα1 direct targets *Ctnnb1* and *Sfrp2* behave in different manner and, surprisingly, their expression is diminished in the double mutant intestines (normal or lesions) compared to the simple *vil*-TRα1 mice (**Figure 1B**). TRα1 and β-catenin/Tcf4 bind to specific genomic sequences called TRE and WNT Responsive Elements (WRE), respectively. We decided to analyze the reciprocal influence of TRα1, β-catenin and Tcf4 in a cellular test *in vitro* using the respective luciferase-driven reporters DR4/TRE (20) and TopFlash/WRE (42). For this aim, we transfected Caco2 cells with the specific reporters and observed that the DR4-luciferase activity dependent on TRα1 can be impaired by the co-transfection of β-catenin and Tcf4 (**Figure 1C**). On the contrary, TRα1 transfection had a small but significant positive effect on the TopFlash-driven luciferase response (**Figure 1C**). All these experiments were conducted in cells in the presence of serum, thus containing physiological concentrations of THs (43). FopFlash and mut-DR4 have been used as negative control and, as expected, we could not detect any modulation of the luciferase activity (data not shown). Intriguingly, the luciferase response was also present in the absence of T3 (our unpublished observation), indicating that TRα1 functionally interacts with the β-catenin/Tcf4 complex independently from the hormone. These results in cells pushed us to investigate the eventual presence of the three proteins on the chromatin, by using an *in vivo* chromatin immunoprecipitation (ChIP) approach to analyze the TREs and WREs of the specific target genes of TRα1 and β-catenin/Tcf4, respectively. As shown in **Figure 1D**, in the WT intestine, TRα1 binds to the promoter region of the *Sfrp2* and *Ctnnb1* genes containing TRE elements (37). As the *Sfrp2* and *Ctnnb1* expression profiles suggested, the TRα1 chromatin occupancy changed between the WT and mutant intestine. In fact, in *vil*-TRα1/Apc mice, the TRα1-specific DNA binding on the regulatory regions is lost, when compared with the WT animals. To analyze the specific DNA binding of β-catenin/Tcf4 on their target genes and the eventual presence of TRα1 in the same regions, we decided to look at WREs described within the *Axin2* (44) and *c-Myc* (45), two classical direct WNT targets. In both cases (**Figure 1D**) TRα1 was not present on the WRE regions in the WT intestine but was clearly enriched on them, both in normal mucosae and the tumors of *vil*-TRα1/Apc mice (**Figure 1D**). As we could not find any TREs within the *Axin2* or *c-Myc* genomic regions in proximity of the WREs analyzed, we speculate that TRα1 might be present as a protein partner to amplify the transcriptional response dependent upon the WNT effectors β-catenin/Tcf4.

## Discussion and Conclusions

**Figure 2:**
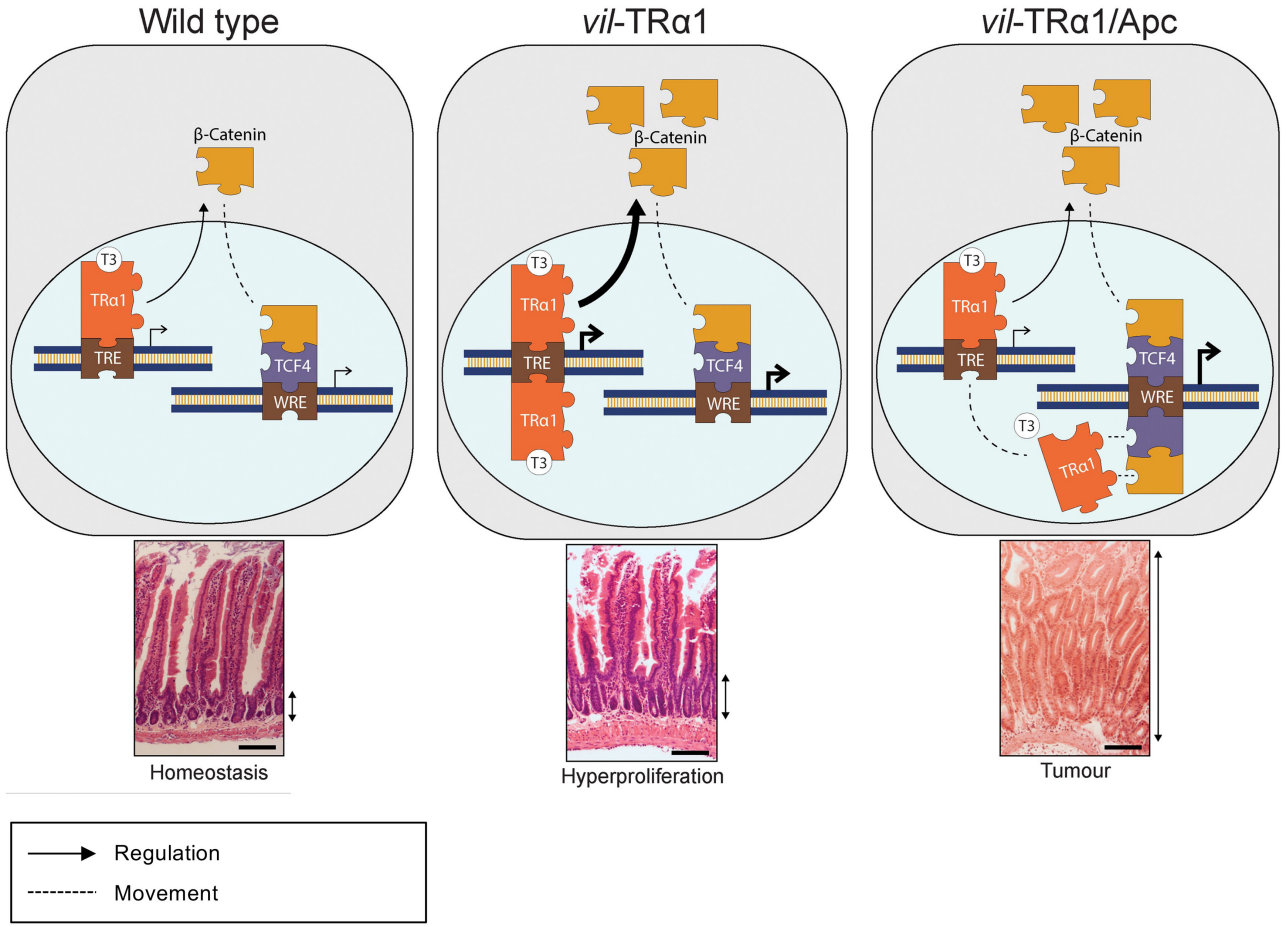
Proposed cross-regulation of TRα1 and Wnt effectors in normal intestine and in tumors. In physiological conditions (Wild Type panel), TRα1 binding on TREs regulates in a positive manner the expression and stabilization of β-catenin, then contributing to maintain epithelial homeostasis. In *vil*-TRα1 mice, the levels of stabilized β-catenin are increased leading to WNT activation and hyper-proliferation. In *vil*-TRα1/Apc mice, the stronger β-catenin stabilization and Tcf4 overexpression might induce a competitive shift in TRα1 binding from TREs to WREs. We can speculate that this mechanism is one of the factors responsible for the activation and/or the acceleration of the tumorigenic process dependent upon TRα1-up regulation. TREs, Thyroid hormone response elements; WREs, WNT response elements. Solid arrows indicate genomic actions; dotted lines represent (i) the β-catenin translocation from cytoplasm to nucleus and (ii) the speculative model of TRα1 shift from TRE to WRE. Thickness of the solid arrows indicates an increased level of transcriptional activity. Double black arrows indicate crypt width in WT and *vil*-TRα1 intestinal sections or the size of the altered mucosa in *vil*-TRα1/Apc tumor.

Complex cross-talks between signaling pathways and hormones, such as T3, have been described in several physiological models and in cancers (41, 46, 47). We reported here recent literature and also included new data on the intriguing relations between THs/TRα1 and WNT/β-catenin pathway in normal intestine and in tumors. From these different findings we propose the model summarized in **Figure 2**. Indeed, in a normal intestine TRα1 and the WNT effectors control their own target genes (Wild Type panel). When TRα1 is up-regulated in a normal context (*vil*-TRα1 mice) it stimulates its target genes including β-catenin, resulting in the activation of the WNT pathway and, by consequence, increased crypt proliferation, through the mechanisms we have already described (41, 48). In the case of the tumors (panel *vil*-TRα1/Apc), the stronger increase of the β-catenin/Tcf4 complex not only induces the WNT pathway activity but may also act to “displace” TRα1 from its own targets to the WNT targets. We may speculate that the “displacement” of TRα1 can be considered the molecular counterpart of the WNT hyperactivation resulting from the interaction of this two signalling pathways. Indeed, in the intestinal tumors of *vil*-TRα1/Apc mice the transcriptional activity of TRα1 on its own target is similar to that observed in WT condition, despite the nuclear receptor is expressed at augmented levels comparable with those of *vil*-TRα1/Apc mice. It is worth noting that TRβ or other nuclear receptors and β-catenin/Tcf (alone or in combination) have been shown to form protein complexes (49–52). We can speculate that TRα1 interacts with β-catenin/Tcf4 complex on WNT targets and that this process might be independent from T3. In addition, this interaction could contribute to the WNT pathway hyper-activation. In the case of the normal mucosae of *vil*-TRα1/Apc mice, the molecular phenotype is even more intriguing, given that these portions of the intestines have histological appearance similar to that of the *vil*-TRα1, but show molecular features close to the tumors.

According to the literature and also considering our work, THs-TRs definitively play a pivotal role in intestinal development and homeostasis in both mouse and amphibians by regulating the expression of a large panel of genes, including those belonging to the WNT pathway and cell cycle regulators (37, 53, 54). It is important to underline that all these studies provide data that can be now used in bioinformatics and statistics analyses for a direct comparison of TH-TR dependent mechanisms in the intestine of different species. Such a study will aim to identify common/uncommon mechanisms, genes directly or indirectly regulated in developmental, homeostatic and pathological conditions. This comparative analysis will surely be of importance for translational research. Indeed, when considering the tumor heterogeneity and cell plasticity within the CRCs (55–57), one of the future challenges will be to fully define the genes and signalling networks influencing the activity of TRα1 and, *vice-versa*, the cascade of regulations depending on THs-TRα1.

## Data Availability Statement

The original contributions presented in the study are included in the article/**Supplementary Material**. Further inquiries can be directed to the corresponding authors.

## Ethics Statement

The animal study was reviewed and approved by Ministère de l’Enseignement Supérieur et de la Recherche, Direction Générale pour la Recherche et l’Innovation, Secrétariat “Autorisation de projet” (agreement #02847.01).

## Author Contributions

MS participated in the design of the study, performed all the experiment, carried out the data analysis and drafted the manuscript. LC helped in design the manuscript, figure preparation and revised the manuscript. MP conceived and supervised the study, carried out the data analysis drafted and revised the manuscript. All authors contributed to the article and approved the submitted version.

## Funding

MP lab is supported by the FRM (Equipes FRM 2018, DEQ20181039598), by the Inca (PLBIO19-289) and by the Ligue Contre le Cancer, Département Grand Est (01X.2020). LC received support from the FRM.

## Conflict of Interest

The authors declare that the research was conducted in the absence of any commercial or financial relationships that could be construed as a potential conflict of interest.

## Publisher’s Note

All claims expressed in this article are solely those of the authors and do not necessarily represent those of their affiliated organizations, or those of the publisher, the editors and the reviewers. Any product that may be evaluated in this article, or claim that may be made by its manufacturer, is not guaranteed or endorsed by the publisher.

## References

[1] Brown DD Cai L. Amphibian Metamorphosis. Dev Biol (2007) 306:20–33. doi: 10.1016/j.ydbio.2007.03.021 17449026 PMC1945045

[2] Gehart H Clevers H. Tales From the Crypt: New Insights Into Intestinal Stem Cells. Nat Rev Gastroenterol Hepatol (2019) 16:19–34. doi: 10.1038/s41575-018-0081-y 30429586

[3] Sailaja BS He XC Li L. The Regulatory Niche of Intestinal Stem Cells. J Physiol (2016) 594:4827–36. doi: 10.1113/JP271931 PMC5009778 27060879

[4] Hryniuk A Grainger S Savory JGA Lohnes D. Cdx Function Is Required for Maintenance of Intestinal Identity in the Adult. Dev Biol (2012) 363:426–37. doi: 10.1016/j.ydbio.2012.01.010 22285812

[5] Perochon J Carroll LR Cordero JB. Wnt Signalling in Intestinal Stem Cells: Lessons From Mice and Flies. Genes (Basel) (2018) 9:1–19. doi: 10.3390/genes9030138 PMC5867859 29498662

[6] Korinek V Barker N Moerer P Van Donselaar E Huls G Peters PJ. Depletion of Epithelial Stem-Cell Compartments in the Small Intestine of Mice Lacking Tcf-4. Nat Genet (1998) 19:379–83. doi: 10.1038/1270 9697701

[7] Kuhnert F Davis CR Wang HT Chu P Lee M Yuan J. Essential Requirement for Wnt Signaling in Proliferation of Adult Small Intestine and Colon Revealed by Adenoviral Expression of Dickkopf-1. Proc Natl Acad Sci USA (2004) 101:266–71. doi: 10.1073/pnas.2536800100 PMC314174 14695885

[8] Pinto D Gregorieff A Begthel H Clevers H. Canonical Wnt Signals Are Essential for Homeostasis of the Intestinal Epithelium. Genes Dev (2003) 17:1709–13. doi: 10.1101/gad.267103 PMC196179 12865297

[9] Van de Wetering M Sancho E Verweij C De Lau W Oving I Hurlstone A. The β-Catenin/TCF-4 Complex Imposes a Crypt Progenitor Phenotype on Colorectal Cancer Cells. Cell (2002) 111:241–50. doi: 10.1016/S0092-8674(02)01014-0 12408868

[10] Ichii S Horii A Nakatsuru S Furuyama J Utsunomiya J Nakamura Y. Inactivation of Both APC Alleles in an Early Stage of Colon Adenomas in a Patient With Familial Adenomatous Polyposis (FAP). Hum Mol Genet (1992) 1:387–90. doi: 10.1093/hmg/1.6.387 1338760

[11] Levy DB Smith KJ Beazer-Barclay Y Hamilton SR Vogelstein B Kinzler KW. Inactivation of Both APC Alleles in Human and Mouse Tumors. Cancer Res (1994) 54:5953–8. 7954428

[12] Fre S Pallavi SK Huyghe M Laé M Janssen KP Robine S. Notch and Wnt Signals Cooperatively Control Cell Proliferation and Tumorigenesis in the Intestine. Proc Natl Acad Sci USA (2009) 106:6309–14. doi: 10.1073/pnas.0900427106 PMC2649205 19251639

[13] Taipale J Beachy PA. The Hedgehog and Wnt Signalling. Nature (2001) 411:349–54. doi: 10.1038/35077219 11357142

[14] Deng F Peng L Li Z Tan G Liang E Chen S. YAP Triggers the Wnt/β-Catenin Signalling Pathway and Promotes Enterocyte Self-Renewal, Regeneration and Tumorigenesis After DSS-Induced Injury. Cell Death Dis (2018) 9(2):153. doi: 10.1038/s41419-017-0244-8 29396428 PMC5833613

[15] Kress E Skah S Sirakov M Nadjar J Gadot N Scoazec JY. Cooperation Between the Thyroid Hormone Receptor Trα1 and the WNT Pathway in the Induction of Intestinal Tumorigenesis. Gastroenterology (2010) 138:1863–74.e1. doi: 10.1053/j.gastro.2010.01.041 20114049

[16] Uchuya-Castillo J Aznar N Frau C Martinez P Le Nevé C Marisa L. Increased Expression of the Thyroid Hormone Nuclear Receptor TRa1 Characterizes Intestinal Tumors With High Wnt Activity. Oncotarget (2018) 9:30979–96. doi: 10.18632/oncotarget.25741 PMC6089551 30123421

[17] Yen PM Ando S Feng X Liu Y Maruvada P Xia X. Thyroid Hormone Action at the Cellular, Genomic and Target Gene Levels. Mol Cell Endocrinol (2006) 246:121–7. doi: 10.1016/j.mce.2005.11.030 16442701

[18] Ishizuya-Oka A Shi YB. Molecular Mechanisms for Thyroid Hormone-Induced Remodeling in the Amphibian Digestive Tract: A Model for Studying Organ Regeneration. Dev Growth Differ (2005) 47:601–7. doi: 10.1111/j.1440-169X.2005.00833.x 16316405

[19] Sirakov M Plateroti M. The Thyroid Hormones and Their Nuclear Receptors in the Gut: From Developmental Biology to Cancer. Biochim Biophys Acta - Mol Basis Dis (2011) 1812:938–46. doi: 10.1016/j.bbadis.2010.12.020 21194566

[20] Plateroti M Kress E Mori JI Samarut J. Thyroid Hormone Receptor α1 Directly Controls Transcription of the β-Catenin Gene in Intestinal Epithelial Cells. Mol Cell Biol (2006) 26:3204–14. doi: 10.1128/mcb.26.8.3204-3214.2006 PMC1446951 16581794

[21] Plateroti M Chassande O Fraichard A Gauthier K Freund JN Samarut J. Involvement of T3Rα- and β-Receptor Subtypes in Mediation of T3 Functions During Postnatal Murine Intestinal Development. Gastroenterology (1999) 116:1376–8. doi: 10.1016/S0016-5085(99)70501-9 10348820

[22] Bao L Roediger J Park S Fu L Shi B Cheng SY. Thyroid Hormone Receptor Alpha Mutations Lead to Epithelial Defects in the Adult Intestine in a Mouse Model of Resistance to Thyroid Hormone. Thyroid (2019) 29:439–48. doi: 10.1089/thy.2018.0340 PMC6437623 30595106

[23] Kress E Rezza A Nadjar J Samarut J Plateroti M. The Thyroid Hormone Receptor-α (Trα) Gene Encoding Trα1 Controls Deoxyribonucleic Acid Damage-Induced Tissue Repair. Mol Endocrinol (2008) 22:47–55. doi: 10.1210/me.2007-0278 17872380 PMC5419624

[24] Godart M Frau C Farhat D Giolito MV Jamard C Le Nevé C. The Murine Intestinal Stem Cells Are Highly Sensitive to the Modulation of the T3/Trα1-Dependent Pathway. Development (2021) 148(8):dev194357. doi: 10.1242/dev.194357 33757992

[25] Krashin E Piekiełko-Witkowska A Ellis M Ashur-Fabian O. Thyroid Hormones and Cancer: A Comprehensive Review of Preclinical and Clinical Studies. Front Endocrinol (Lausanne) (2019) 10:59. doi: 10.3389/fendo.2019.00059 30814976 PMC6381772

[26] Perra A Plateroti M Columbano A. T3/TRs Axis in Hepatocellular Carcinoma: New Concepts for an Old Pair. Endocr Relat Cancer (2016) 23:R353–69. doi: 10.1530/ERC-16-0152 27353037

[27] Hörkkö TT Tuppurainen K George SM Jernvall P Karttunen TJ Mäkinen MJ. Thyroid Hormone Receptor β 1 in Normal Colon and Colorectal Cancer – Association With Differentiation, Polypoid Growth Type and K-Ras Mutations. Int J Cancer (2006) 118:1653–9. doi: 10.1002/ijc.21556 16231318

[28] Brown AR Simmen RCM Simmen FA. The Role of Thyroid Hormone Signaling in the Prevention of Digestive System Cancers. Int J Mol Sci (2013) 14(8):16240–57. doi: 10.3390/ijms140816240 PMC3759909 23924944

[29] Cristofanilli M Yamamura Y Kau SW Bevers T Strom S Patangan M. Thyroid Hormone and Breast Carcinoma: Primary Hypothyroidism Is Associated With a Reduced Incidence of Primary Breast Carcinoma. Cancer (2005) 103:1122–8. doi: 10.1002/cncr.20881 15712375

[30] Moeller LC Führer D. Thyroid Hormone, Thyroid Hormone Receptors, and Cancer: A Clinical Perspective. Endocr Relat Cancer (2013) 20(2):R19–29. doi: 10.1530/ERC-12-0219 23319493

[31] Boelen A Kwakkel J Fliers E. Beyond Low Plasma T 3: Local Thyroid Hormone Metabolism During Inflammation and Infection. Endocr Rev (2011) 32:670–93. doi: 10.1210/er.2011-0007 21791567

[32] Bianco AC Kim BW. Deiodinases: Implications of the Local Control of Thyroid Hormone Action. J Clin Invest (2006) 116(10):2571–9. doi: 10.1172/JCI29812 PMC1578599 17016550

[33] Dentice M Luongo C Huang S Ambrosio R Elefante A Mirebeau-prunier D. Sonic Hedgehog-Induced Type 3 Deiodinase Blocks Thyroid Hormone Action Enhancing Proliferation of Normal and Malignant Keratinocytes. Proc Natl Acad Sci USA (2007) 104:14466–71. doi: 10.1073/pnas.0706754104 PMC1964817 17720805

[34] Dentice M Luongo C Ambrosio R Sibilio A Casillo A Iaccarino A. β-Catenin Regulates Deiodinase Levels and Thyroid Hormone Signaling in Colon Cancer Cells. Gastroenterology (2012) 143:1037–47. doi: 10.1053/j.gastro.2012.06.042 22771508

[35] Catalano V Dentice M Ambrosio R Luongo C Carollo R Benfante A. Activated Thyroid Hormone Promotes Differentiation and Chemotherapeutic Sensitization of Colorectal Cancer Stem Cells by Regulating Wnt and BMP4 Signaling. Cancer Res (2016) 76:1237–44. doi: 10.1158/0008-5472.CAN-15-1542 26676745

[36] Markowitz S Haut M Stellato T Gerbic C Molkentin K. Rapid Publication Expression of the ErbA-F3 Class of Thyroid Hormone Receptors Is Selectively Lost in Human Colon Carcinoma. J Clin Invest (1989) 84(5):1683–7. doi: 10.1172/JCI114349 PMC304038 2553781

[37] Kress E Rezza A Nadjar J Samarut J Plateroti M. The Frizzled-Related Sfrp2 Gene is a Target of Thyroid Hormone Receptor α1 and Activates β-Catenin Signaling in Mouse Intestine. J Biol Chem (2009) 284:1234–41. doi: 10.1074/jbc.M806548200 19001373

[38] Bovolenta P Esteve P Ruiz JM Cisneros E Lopez-Rios J. Beyond Wnt Inhibition: New Functions of Secreted Frizzled-Related Proteins in Development and Disease. J Cell Sci (2008) 121:737–46. doi: 10.1242/jcs.026096 18322270

[39] Skah S Nadjar J Sirakov M Plateroti M. The Secreted Frizzled-Related Protein 2 Modulates Cell Fate and the Wnt Pathway in the Murine Intestinal Epithelium. Exp Cell Res (2015) 330:56–65. doi: 10.1016/j.yexcr.2014.10.014 25447442

[40] Fodde R Edelmann W Yang K Van Leeuwen C Carlson C Renault B. A Targeted Chain-Termination Mutation in the Mouse Apc Gene Results in Multiple Intestinal Tumors. Proc Natl Acad Sci USA (1994) 91:8969–73. doi: 10.1073/pnas.91.19.8969 PMC44728 8090754

[41] Skah S Uchuya-Castillo J Sirakov M Plateroti M. The Thyroid Hormone Nuclear Receptors and the Wnt/β-Catenin Pathway: An Intriguing Liaison. Dev Biol (2017) 422:71–82. doi: 10.1016/j.ydbio.2017.01.003 28069375

[42] Van de Wetering M Cavallo R Dooijes D Van Beest M Van Es J Loureiro J. Armadillo Coactivates Transcription Driven by the Product of the Drosophila Segment Polarity Gene dTCF. Cell (1997) 88:789–99. doi: 10.1016/S0092-8674(00)81925-X 9118222

[43] Samuels HH Stanley F C J. Depletion of L-3,5,39-Triiodothyronine and L-Thyroxine in Euthyroid Calf Serum for Use in Cell Culture Studies of the Action of Thyroid Hormone. Endocrinology (1979) 105:80–5. doi: 10.1210/endo-105-1-80 446419

[44] Mahmoudi T Boj SF Hatzis P Li VSW Taouatas N Vries RGJ. The Leukemia-Associated Mllt10/Af10-Dot1l Are Tcf4/β-Catenin Coactivators Essential for Intestinal Homeostasis. PloS Biol (2010) 8(11):e1000539. doi: 10.1371/journal.pbio.1000539 21103407 PMC2982801

[45] Hu MC Rosenblum ND. Smad1, β-Catenin and Tcf4 Associate in a Molecular Complex With the Myc Promoter in Dysplastic Renal Tissue and Cooperate to Control Myc Transcription. Development (2005) 132:215–25. doi: 10.1242/dev.01573 15576399

[46] Garg D Ng SSM Baig KM Driggers P Segars J. Progesterone-Mediated Non-Classical Signaling. Trends Endocrinol Metab (2017) 28:656–68. doi: 10.1016/j.tem.2017.05.006 28651856

[47] Ni F-D Hao S-L Yang W-X. Molecular Insights Into Hormone Regulation *via* Signaling Pathways in Sertoli Cells: With Discussion on Infertility and Testicular Tumor. Gene (2020) 753:144812. doi: 10.1016/j.gene.2020.144812 32470507

[48] Sirakov M Kress E Nadjar J Plateroti M. Thyroid Hormones and Their Nuclear Receptors: New Players in Intestinal Epithelium Stem Cell Biology? Cell Mol Life Sci (2014) 71:2897–907. doi: 10.1007/s00018-014-1586-3 PMC11113153 24604390

[49] Pakula H Xiang D Li Z. A Tale of Two Signals: AR and WNT in Development and Tumorigenesis of Prostate and Mammary Gland. Cancers (Basel) (2017) 9:1–34. doi: 10.3390/cancers9020014 PMC5332937 28134791

[50] Cao J Ma Y Yao W Zhang X Wu D. Retinoids Regulate Adipogenesis Involving the Tgfβ/SMAD and Wnt/β-Catenin Pathways in Human Bone Marrow Mesenchymal Stem Cells. Int J Mol Sci (2017) 18(4):842. doi: 10.3390/ijms18040842 PMC5412426 28420144

[51] Reinhold S Blankesteijn WM Foulquier S. The Interplay of WNT and Pparγ Signaling in Vascular Calcification. Cells (2020) 9:1–27. doi: 10.3390/cells9122658 PMC7763279 33322009

[52] Muralidhar S Filia A Nsengimana J Pozniak J O’Shea SJ Diaz JM. Vitamin D–VDR Signaling Inhibits Wnt/b-Catenin–Mediated Melanoma Progression and Promotes Antitumor Immunity. Cancer Res (2019) 79:5986–98. doi: 10.1158/0008-5472.CAN-18-3927 31690667

[53] Hasebe T Fujimoto K Kajita M Ishizuya-Oka A. Thyroid Hormone Activates Wnt/β-Catenin Signaling Involved in Adult Epithelial Development During Intestinal Remodeling in Xenopus Laevis. Cell Tissue Res (2016) 365:309–18. doi: 10.1007/s00441-016-2396-8 27068920

[54] Shibata Y Tanizaki Y Zhang H Lee H Dasso M Shi Y-B. Thyroid Hormone Receptor Is Essential for Larval Epithelial Apoptosis and Adult Epithelial Stem Cell Development But Not Adult Intestinal Morphogenesis During Xenopus Tropicalis Metamorphosis. Cells (2021) 11(9):1368. doi: 10.3390/cells10030536 PMC8000126 33802526

[55] Teeuwssen M Fodde R. Cell Heterogeneity and Phenotypic Plasticity in Metastasis Formation: The Case of Colon Cancer. Cancers (Basel) (2019) 11(9):1368. doi: 10.3390/cancers11091368 PMC6770401 31540068

[56] Zeuner A Todaro M Stassi G De Maria R. Colorectal Cancer Stem Cells: From the Crypt to the Clinic. Cell Stem Cell (2014) 15:692–705. doi: 10.1016/j.stem.2014.11.012 25479747

[57] Hirata A Hatano Y Niwa M Hara A T H. Heterogeneity of Colon Cancer Stem Cells. In: Birbrair A, editor. Stem Cells Heterogeneity in Cancer. Advances in Experimental Medicine and Biology. Cham: Springer. p. 1139:115–26. doi: 10.1007/978-3-030-14366-4_7 31134498

